# Culture dependent and independent characterization of endophytic bacteria in the seeds of highland barley

**DOI:** 10.3389/fmicb.2022.981158

**Published:** 2022-09-23

**Authors:** Yulan Chen, Jinpeng Liang, Alina Zia, Xue Gao, Yong Wang, Lingzi Zhang, Quanju Xiang, Ke Zhao, Xiumei Yu, Qiang Chen, Petri Penttinen, Tashi Nyima, Yunfu Gu

**Affiliations:** ^1^Department of Microbiology, College of Resources, Sichuan Agricultural University, Chengdu, China; ^2^Liangshan Tobacco Corporation of Sichuan Province, Xichang, China; ^3^Institute of Agricultural Resources and Environmental Science, Tibet Academy of Agricultural and Animal Husbandry Sciences, Lhasa, Tibet, China

**Keywords:** Highland barley, endophytic bacteria, pure culture, 16S rRNA gene sequencing, plant growth promoting bacteria

## Abstract

Endophytes in the seeds of plants have shown plant growth promoting (PGP) properties. Highland barley is an economically important crop and a major part of the local diet in the Tibetan Plateau, China, with potential health benefits. We applied culture-dependent and culture-independent methods to study endophytic bacteria in the seeds of eight Highland barley varieties. Based on the seed properties, the variety Ali was clearly separated from the other varieties except the variety CM. Most of the 86 isolates were assigned into genus Bacillus. Approximately half of the isolates showed PGP properties *in vitro*. Compared to the not-inoculated plants, inoculation with the isolate *Bacillus tequilensis* LZ-9 resulted in greater length and number of roots, and in bigger aboveground and root weights. Based on the 16S rRNA gene sequencing, the seed microbiome was majorly affiliated with the phylum Proteobacteria and the family Enterobacteriaceae. Overall, the bacterial community compositions in the different varieties were different from each other, yet the between variety differences in community composition seemed relatively small. The differences in community compositions were associated with differences in the total and reducing sugar contents and viscosity of the seeds, thus possibly connected to differences in the osmotic pressure tolerance of the endophytes. The results suggested that the seed endophytes are likely to promote the growth of Highland barley since germination.

## Introduction

Plants form associations with a multitude of structurally and functionally diverse beneficial microbes that provide them selective advantages. The beneficial associates include endophytic bacteria, i.e., non-pathogenic bacteria that reside within the living tissues of plants without conferring them harm. Exploring the relationships between plants and their microbiomes is a hot topic in ecology, plant sciences, and agronomy (Vandenkoornhuyse et al., 2015). Plant compartments (Wang et al., 2016), genotypes, and geographic locations (Edwards et al., 2015) are vital factors in shaping the endophytic microbiome composition. Mora-Ruiz et al. (2016) found that the internal tissues of *Arthrocnemum macrostachyum* can serve as a suitable environment for the colonization of moderately halophilic bacteria. Furthermore, the seed endophytic community of different rice cultivars varied with the genotype (Walitang et al., 2018), and the endophytic fungal community composition in *Elymus nutans* seeds varied with geographical location (Guo et al., 2021). Although the importance of plant endophytic microbes in plant growth and health is getting more recognition, the role of seed-associated microorganisms, especially seed endophytic bacteria, is still not well understood.

Seeds represent a remarkable phase in the life cycle of spermatophytes: they can persist for years in a dormant state and, when the appropriate conditions are met, develop into a new plant (Nelson, 2004). Diverse microorganisms in plant seeds are critical to seed and plant health (Grumet and Gifford, 1998; Gitaitis and Walcott, 2007). Plant endophytes were first discovered in ryegrass seeds by in Vogl (1989), and the importance of seed bacteria has gradually been realized in recent decades. Although it is estimated that only 0.001–1% of endophytic bacteria can be cultivated (Eevers et al., 2015), the knowledge on seed endophytic microbes is constantly expanding due to the application of cultivation-independent techniques. e.g., high-throughput sequencing (Verma et al., 2017; Durand et al., 2021). Multi-omics techniques have significantly improved the understanding of the role of the seed endophytic microbiome. The isolated seed endophytes have been mostly Proteobacteria, especially *γ*-Proteobacteria, followed by the Actinobacteria, Firmicutes, and Bacteroidetes phyla (Truyens and Cuypers, 2015). In general, *Bacillus, Pseudomonas, Paenibacillus, Micrococcus, Staphylococcus, Pantoea*, and *Acinetobacter* genera have been often detected in the seeds. The seed endophytes may be passed on to the next generation; rice seeds hosted bacteria that were detected in roots and stems after germination (Kaga et al., 2009), and a core microbiome was detected in *Crotalaria pumila* seeds over three years (Sánchez-López et al., 2018). The endophytes in the seeds of barley and other plants have shown PGP properties both *in vitro* and *in vivo* (Rahman et al., 2018; Sánchez-López et al., 2018; Rios-Galicia et al., 2021). Seed endophytes with PGP properties are desirable in agriculture because they are likely to promote the plant growth since germination and act before the soil-borne PGP bacteria (Mitter et al., 2017).

Highland barley (*Hordeum vulgare* L.var. *nudum* hook. f, HB), one of the cereal grasses in the Gramineae family, is also called hull-less barley or naked barley because it’s inner and outer glumes are separated from caryopsis when harvested (Gao et al., 2015; Deng et al., 2018). HB is mainly grown in Qinghai-Tibet Plateau in China on an approximately 0.27 million hectares planting area that accounts almost 90% of all HB production (Guo et al., 2020). Recently, HB has received interest due to its potential health benefits (Obadi et al., 2021).

To our knowledge, the endophytes in HB seeds have not been studied to date. We applied culture-dependent and culture-independent methods to determine (1) the identities and plant growth promoting (PGP) properties of the culturable endophytic bacteria in HB seeds; (2) the composition of endophytic bacterial communities in HB seeds; and (3) the relationships between the endophytic bacterial communities and HB seed properties. We hypothesized that (i) HB seeds harbor culturable endophytic bacteria with PGP properties, and (ii) the composition of the endophytic bacterial communities in HB seeds is related to the seed properties.

## Materials and methods

### Properties of the highland barley varieties

**Figure 1:**
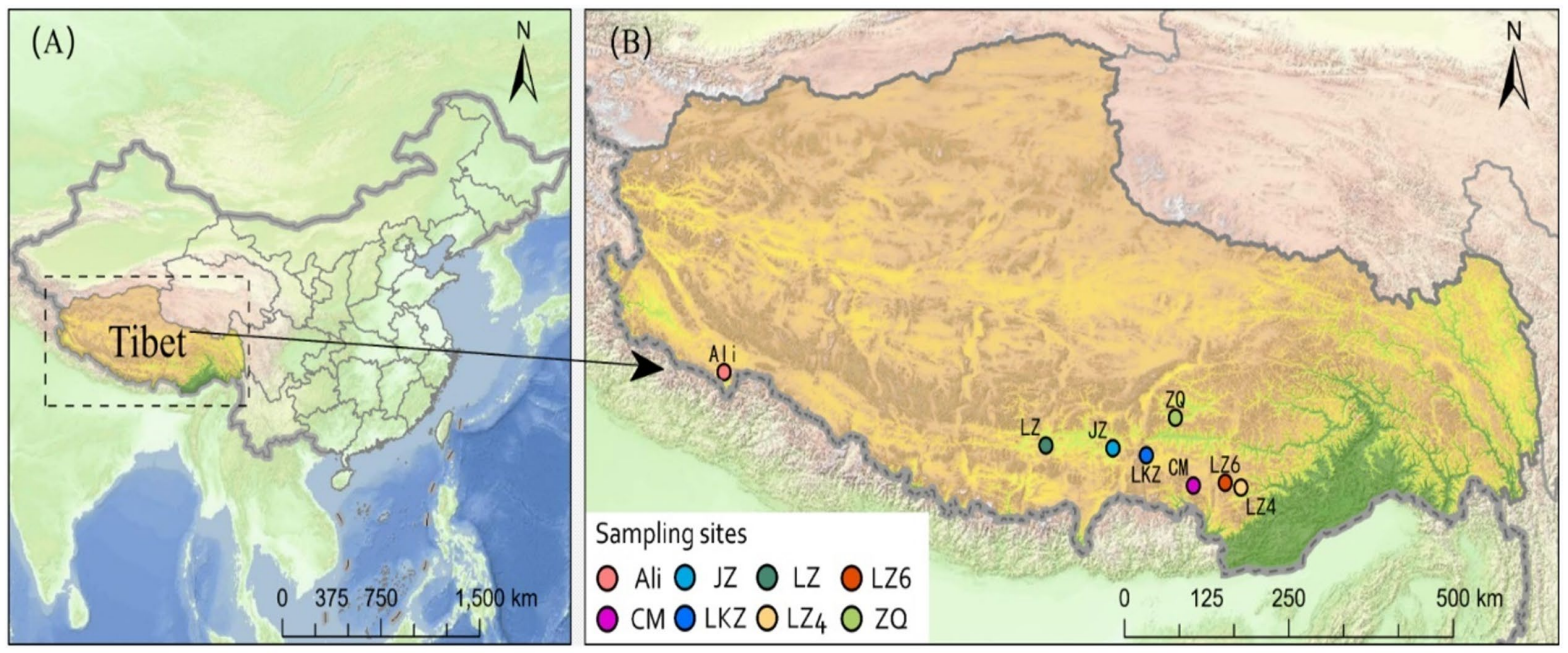
The locations of sampling sites of the eight highland barley varieties in Tibet Autonomous Region, China. CM, Cuomei; JZ, Jiangzi; LZ, Lazi; LZ4, Longzi4ling; LZ6, Longzi6ling; LKZ, Langkazi; and ZQ, Zangqing 2000.

In September 2020, highland barley (HB) varieties Cuomei (CM), Jiangzi (JZ), Lazi (LZ), Ali, Zangqing 2000 (ZQ), Longzi4ling (LZ4), Longzi6ling (LZ6), and Langkazi (LKZ) were collected from eight sites in Tibet Autonomous Region, China (Figure 1). At each site, five plots of 10 m × 10 m were randomly established, and each plot was further divided into four 5 m × 5 m subplots. In each subplot, HB seeds were collected from different plant individuals. At the laboratory, the seeds were dried in a desiccator with silica gel desiccant cartridges, then immediately brought to −20°C. Three 500 g replicates were made from each seed stock. Details on the altitude and soil physico-chemical properties of the sites are in Supplementary Table S1.

The genetic polymorphism of the eight HB varieties was assessed by analyzing the simple sequence repeats (SSRs) as described by Liu et al. (1996). Amylose content (AM) in the HB seeds was determined using colorimetric method No 61–03 at 620 nm (AACC, 2000). Total and reducing sugars (TS and RS) contents were analyzed following anthrone and Nelson-Somogyi methods, respectively (Thomas and James, 1999; AACC, 2000). Washed gluten was kept in a shaking water bath at 37°C for different time intervals, after which wet gluten (WG) content was determined using standard methods (AACC, 2000). Sedimentation value (SV) was determined using Zeleny method, soluble protein content (SP) was determined using Kjeldahl method, and viscosity (RVA) was analyzed using Rapid Visco Analyser (RVA, Newport Science Corp. Australia; AACC, 2000). Average 1,000-kernel weight (TKW) was determined by weighing two 500 kernel samples, averaging the values and multiplicating by 2. All the analyses were done in three replicates.

### Isolation and identification of endophytic bacteria

Endophytic bacteria were isolated as described by Huang and Pang (2017). Ten grams of seeds were surface-sterilized by immersing in 10% sodium hypochlorite for 10 min, rinsed six times with sterile distilled water, kept in 75% ethanol for 1 min, and rinsed three times with distilled water, after which the sterilization procedure was repeated. 100 μl of the final rinse water was inoculated onto Luria-Bertani (LB) agar plates; sterilization was considered successful when no colonies appeared after incubation at 30°C for 48 h. Five grams of sterilized seeds were mashed aseptically in 10 ml PBS buffer for 1 min, followed by centrifugation at 10,000 rpm for 2 min. The supernatant was diluted and inoculated onto LB plates (pH 7.0) that were incubated at 30°C for 48–72 h. Single colonies were picked and purified by streaking repeatedly on fresh LB plates.

Genomic DNA of the endophytic bacteria was isolated using the QIAmp DNA mini kit (Qiagen, Carlsbad, CA, United States) following manufacturer’s protocol. The 16S rRNA genes of the isolates were amplified with primers 27-F (5′-AGAGTTTGATCCGGCTCAG-3′) and 1,492-R (5′-GGTTACC- TTGTTACGACTT-3′; DeLong, 1992). The PCR procedure included initial denaturation at 94°C for 4 min, followed by 35 cycles with denaturation at 94°C for 30 s, annealing at 55°C for 40 s and extension at 72°C for 60 s, and a final extension for 7 min at 72°C. The PCR products were purified and used for ARDRA analysis as described previously (Vaneechoutte et al., 1992). The PCR products with different ARDRA patterns were sequenced at Shengong Biotechnology Ltd. (Shanghai, China). The sequences were identified using BLAST against the NCBI database. A phylogenetic tree was constructed using neighbor-joining method in MEGA 6.0 (Tamura et al., 2013). The sequences were deposited in the NCBI GenBank under the accession numbers MW881426–MW881449 and MZ148645–MZ148646.

### Plant growth-promoting properties

The potential plant growth-promoting (PGP) abilities of the isolates were evaluated *in vitro* and *in vivo*. IAA production was estimated as described by Glickmann and Dessaux (1995). Briefly, isolates were grown in liquid LB medium (with 2 mg ml^−1^ l-tryptophan) at 30°C for 48 h in a shaker (150 rpm). Then, cultures were centrifuged at 10,000 *g* for 30 min and 1 ml the supernatant was mixed with 2 ml Salkowski reagent (1 ml of 0.5 M FeCl_3_ and 49 ml of 35% HClO_4_). After reacting for 0.5 h at room temperature, the IAA concentration was determined at 530 nm by UV–VIS spectrophotometry and calculated based on the standard curve of IAA ranging from 1 to 50 μg ml^−1^. The phosphorus-solubilizing activity of the isolates were qualitatively screened as described by Pikovskaya (1948): the isolates were inoculated onto Pikovskaya’s agar, cultured at 28°C for 5 days, and the isolates with clear halo zone around the colony were considered phosphorus-solubilizing. Cellulase assay was done as described previously (Teather and Wood, 1982). The isolates were grown on M9 medium (Qiagen, Carlsbad, CA, United States) containing 0.5% yeast extract and 1% carboxymethylcellulose (CMC). After 4 days at 30°C, 10 ml of Congo red dye (1%) was added and the plates were washed with 5 M NaCl. Both phosphorus-solubilizing and cellulase activities were evaluated according to the ratio of the halo diameter (HD, in mm) to the colony diameter (CD, in mm) of bacterial isolates on the relevant culture medium (HD/CD). Ability to fix nitrogen was firstly determined in nitrogen-free NFb liquid medium as described by Döbereiner et al. (1995) where a color change from green to blue is indicative of nitrogen fixation; nitrogen fixation was later verified using the acetylene reduction assay (Hardy et al., 1968). Siderophore production was assayed on chrome azurol S (CAS) agar (Schwyn and Nielands, 1987). The isolates were cultured at 28°C for 2 days, and an orange halo around a colony was indicative of siderophore production. All assays were done in triplicate.

The isolates with highest PGP activities *in vitro*, Zang8, JZ-7, and LZ6-9, were selected for further experiments to test their effects on plant growth in greenhouse. Quartz sand (particle size 1 mm) was sterilized at 121°C for 2 h. The seeds of HB variety Zangqing-2000 were surface-sterilized with 70% ethanol and 30% H_2_O_2_ (1:1) for 20 min and washed with ddH_2_O. Seed sterility was verified by incubating 10 seeds on LB agar at 25°C for 4 days. The bacterial isolates were cultivated in LB liquid medium for 24 h at 28°C, centrifuged, washed, and resuspended to a density of 4 × 10^7^ cfu ml^−1^ in ddH_2_O. After the seeds had been germinated in the dark for 2 days, the seeds were soaked for 2 h in the bacterial suspension or sterile water as an uninoculated control. Five seeds per pot were planted in plastic pots filled with 500 g sand. The pots were watered with approximately 15 ml Hoagland’s nutrient solution every day. After 45 days, the plants were carefully removed from the pots, the roots and above-ground parts were separated and washed with distilled water, and dry weights were determined by drying to constant weight. The *in vivo* PGP activities were assayed in three replicates.

### 16S rRNA gene amplicon sequencing of the endophyte community

Amplicon sequencing targeting the 16S rRNA gene was used to characterize the endophytic bacterial communities in the seeds of un-inoculated HB varieties. Immediately after surface sterilization, seeds were dried at ambient temperature in sterile conditions under a laminar flow hood. The dry seeds were ground into a homogenous powder with a Mixer Mill for 30 s at 30 Hz (model MM400; Retsch Inc., Newtown, Pennsylvania, United States) and 5 mm zirconium oxide beads in sterile conditions. Total DNA was extracted using a modified hexadecyltrimethyl ammonium bromide (CTAB) chloroform protocol (Healey et al., 2014). The sterilized seeds were incubated 1 h at 65°C with agitation in the CTAB buffer (2 g CTAB, 4 ml 0.5 M EDTA, 10 ml 1 M Tris–HCl, and 86 ml 1.4 M NaCl in 100 ml), followed by a heat shock treatment from −80 to 65°C and enzymatic digestions with proteinase K, *α*-amylase, and RNAase A. The DNA was firstly precipitated with isopropanol and then washed with 70% ethanol at room temperature twice. The final purification was done using the QIAquick® PCR Purification Kit (Qiagen, Carlsbad, CA, United States). The quantity and quality of DNA were assessed using 1% agarose gel electrophoresis and a SmartSpec™ Plus spectrophotometer (BIO-RAD, United States).

The 16S rRNA gene V4 hypervariable region was amplified with primers 515F (5′-GTGCCAGC- MGCCGCGGTAA-3′) and 806R (5′-GGACTACVSGGGTATCTAAT-3′) with adapter and barcode sequences (Caporaso et al., 2012). Amplification was done in a 50 μl reaction mixture with 3 U of TaKaRa Ex Taq HS (TaKARA Shuzo Co., Shiga, Japan), 5 mM dNTP mixture (TaKARA), 2.0 mM MgCl_2_, 5 μl of 10 × Ex Taq Buffer (TaKARA), 0.6 μM of each primer, and 4.0 ng of DNA. The PCR procedure included initial denaturation at 94°C for 4 min, 30 cycles of 15 s at 94°C, 15 s at 55°C and 30 s at 72°C, and a final extension at 72°C for 10 min. PCR products were purified using PCR Clean-up Purification Kit (MP Biomedicals, Solon, OH, United States) and quantified using Qubit 2.0 fluorimeter (Invitrogen, Carlsbad, CA, United States). Purified amplicons were pooled in equimolar concentrations and sequenced using MiSeq Reagent Kit V2 on an Illumina MiSeq sequencer (MiSeq, Illumina Inc., San Diego, CA, United States). The sequence data were submitted to NCBI Sequence Read Archive^1^ with accession number PRJNA637532.

### Bioinformatics and statistical analysis

Sequence reads were processed with QIIME2 v2019.4 (Bokulich et al., 2018) according to the official tutorials.^2^ Briefly, the raw sequence data were demultiplexed using the demux plugin followed by primer cutting with the QIIME2 Cutadapt plugin (Martin, 2011). QIIME’s split_libraries_fastq.py script was used for quality filtering: reads with Phred quality score < 29 and consecutive, high-quality base calls less than 90% of the read’s length were discarded. Chimeric, singleton, and non-bacterial sequences such as chloroplast and mitochondrial sequences were removed with the debulr plugin (Schuler et al., 2016). Nonsingleton amplicon sequence variants (ASVs) were aligned with mafft (Katoh et al., 2002). After rarefying, Shannon diversity index was estimated using the diversity plugin in QIIME2. Taxonomy was assigned to the ASVs using the classify-sklearn naïve Bayes taxonomy classifier in feature-classifier plugin against the Greengenes database v13.8 (DeSantis et al., 2006).

Differences in the seed properties, *in vivo* PGP properties and alpha diversities of the seed bacterial communities were tested with one-way ANOVA in the Statistical Package for the Social Sciences (SPSS 19.0, SPSS Inc., Chicago, IL, United States). Variation in the seed properties were visualized using principal component analysis (PCA) in CANOCO 4.5 software (CANOCO, Microcomputer Power Inc., Ithaca, NY, United States). Beta diversity was estimated using Hellinger transform based Bray–Curtis dissimilarities (Legendre and Gallagher, 2001), visualized using principal coordinates analysis (PCoA), and tested using Permutational Multivariate ANOVA (PERMANOVA) with the “adonis” function in the R package vegan in R version 3.6.1 (Oksanen et al., 2017). The relationships between bacterial community structure and standardized seed properties were tested using dbRDA and Mantel test in R package vegan and visualized using R packages ggplot2 v3.3.5 and ggrepel v0.9.1 (Slowikowski et al., 2018). Spearman correlations between the abundance of bacteria taxa and seed properties were calculated in the R package WGCNA v1.70–3 (Langfelder and Horvath, 2008).

## Results

### Properties of the highland barley varieties

**Figure 2:**
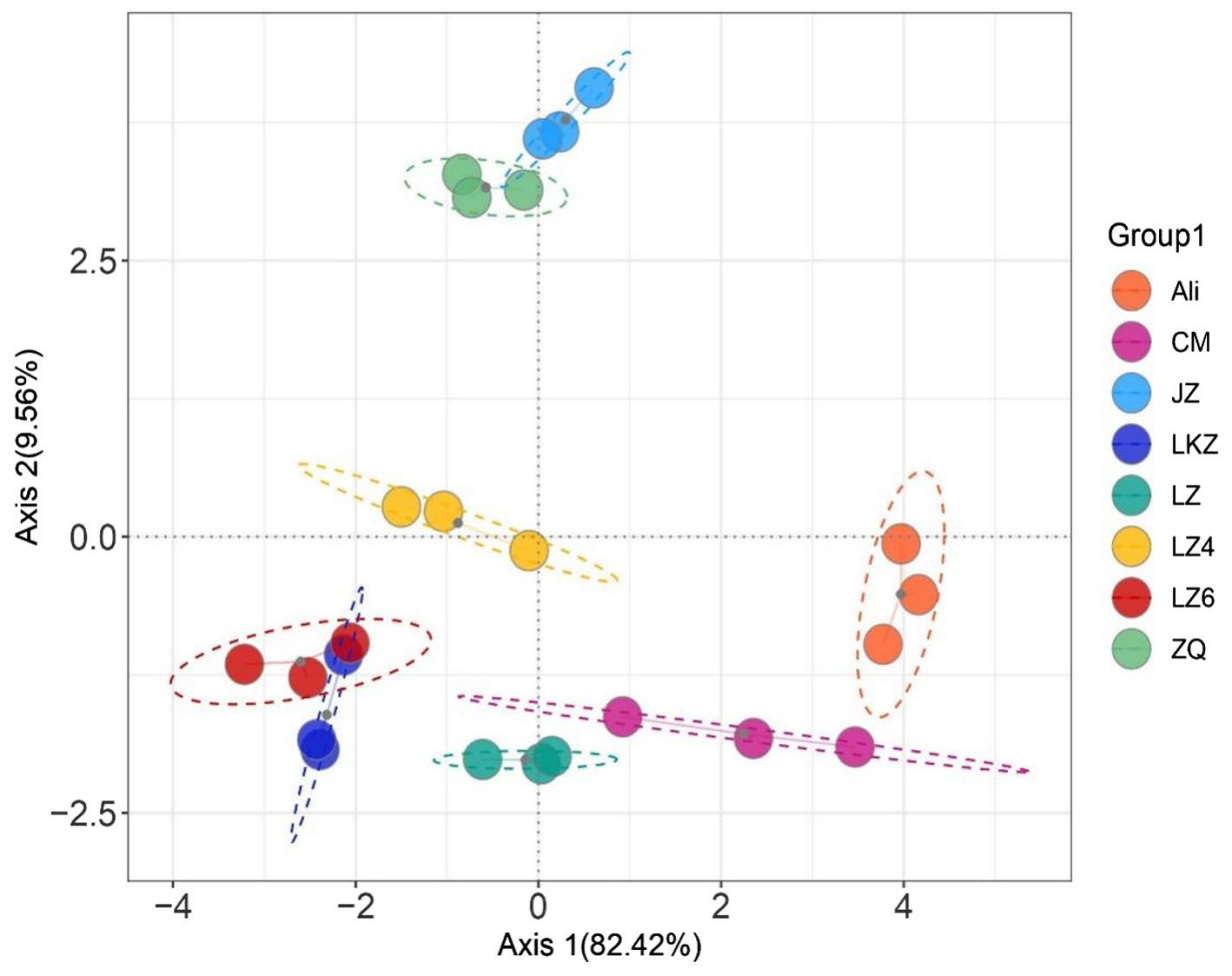
Principal component analysis (PCA) based on the seed properties of the highland barley varieties. CM, Cuomei; JZ, Jiangzi; LZ, Lazi; LZ4, Longzi4ling; LZ6, Longzi6ling; LKZ, Langkazi; and ZQ, Zangqing 2000.

The average similarity coefficient index of the eight highland barley varieties was 0.72 in the ISSR molecular marker analysis (Supplementary Table S2). Based on the seed properties (Table 1), the variety Ali was clearly separated from the other varieties except the variety CM in the principal component analysis (PCA; Figure 2).

**Table 1:** The properties of the eight HB varieties from Tibet Plateau, China.

Samples	Am (%)	TS (%)	RS (%)	WG (%)	SV (ml)	SP (%)	RVA (RVA units)	TKW (g)
LZ	57.9 ± 0.125c	72.9 ± 1.06b	4.80 ± 0.135b	9.10 ± 0.294a	25.1 ± 0.125b	11.6 ± 0.360ab	213 ± 1.69b	46.9 ± 0.472a
Ali	62.2 ± 0.529b	84.7 ± 0.356a	11.4 ± 0.216a	8.91 ± 0.116a	22.0 ± 0.098c	12.7 ± 0.260a	250 ± 0.902a	48.5 ± 1.11a
CM	55.8 ± 0.191b	83.2 ± 0.402a	6.31 ± 0.215b	5.39 ± 0.065c	18.3 ± 0.166d	11.4 ± 0.303ab	234 ± 5.62ab	44.8 ± 0.702a
JZ	70.7 ± 0.659a	82.0 ± 0.223a	5.29 ± 0.077b	6.69 ± 0.222b	29.5 ± 0.314a	9.17 ± 0.291b	217 ± 1.27b	36.9 ± 0.262b
LZ4	57.4 ± 0.899b	77.2 ± 0.072ab	4.61 ± 0.113b	9.47 ± 0.177a	24.7 ± 0.152b	11.2 ± 0.307ab	207 ± 3.28b	34.7 ± 0.538b
LZ6	57.2 ± 0.294b	76.7 ± 0.706ab	5.35 ± 0.075b	9.38 ± 0.332a	21.0 ± 0.406c	11.7 ± 0.152ab	190 ± 2.52c	38.0 ± 0.354b
LKZ	60.9 ± 0.715b	83.1 ± 0.276a	5.34 ± 0.129b	8.76 ± 0.209a	20.3 ± 0.191c	11.2 ± 0.072ab	191 ± 0.766c	47.5 ± 0.648a
ZQ	70.1 ± 0.519a	82.4 ± 0.098a	5.46 ± 0.102b	6.22 ± 0.267b	22.3 ± 0.378c	10.1 ± 0.035b	209 ± 1.06b	35.2 ± 0.470b

Data are mean value ± SE (*n* = 3). Different letters in a column denote statistically significant differences (*p* < 0.05). AM, Amylose content; TS, total sugar; RS, reducing sugar; WG, wet gluten; SV, sedimentation value; SP, soluble protein; RVA, viscosity; and TKW, average 1,000-kernel weight.

### ARDRA and phylogenetic analysis

**Figure 3:**
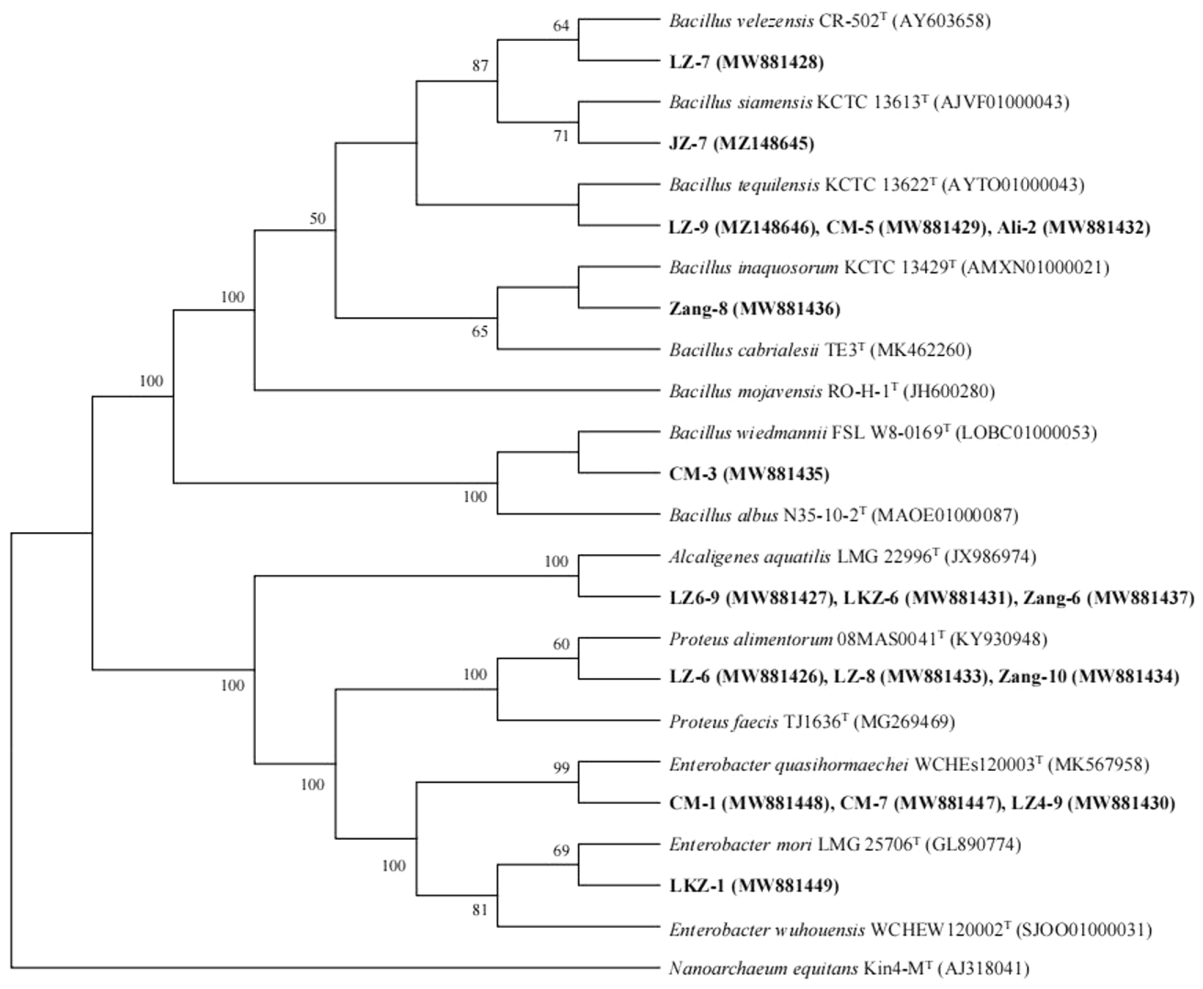
Relationships between the representative isolates and reference strains based on 16S rRNA gene (~1,500 bp) phylogenetic analysis. *Nanoarchaeum equitans* Kin4-M^T^ (AJ318041) was used as an outgroup. Sequences from this study are in bold. Bootstrap values over 50% are shown on the branching points.

In total, 86 bacterial strains were isolated from the seeds of the eight HB varieties. In estimating the genetic diversity of the isolates, the isolates clustered into 17 ARDRA types (Supplementary Table S3). Based on the 16S rRNA gene sequences of representative ARDRA type isolates, the isolates represented one Gram-positive and three Gram-negative genera. Seven ARDRA types were identified as *Bacillus* spp. (Figure 3; Supplementary Table S3). The isolates LZ-9, CM-5, and Ali-2 were closely related to *Bacillus tequilensis*, and LZ-7, JZ-7, Zang-8, and CM-3 were 99.85–99.93% similar with the type strains of *B. velezensis*, *B. siamensis*, *B. inaquosorum*, and *B. wiedmannii*, respectively. LZ6-9, LKZ-6, and Zang-6 were identified as *Alcaligenes aquatilis*, LZ-6, LZ-8, and Zang-10 as *Proteus alimentorum*, and CM-7, CM-1, LZ4-9, and LKZ-1 as *Enterobacter* spp.

### Plant growth-promoting properties

Among the 86 isolates, 41 were positive for at least one and nine isolates for all the three tested plant-growth promoting properties *in vitro* (Supplementary Table S4). Fifty-one isolates produced IAA at concentrations ranging from 3.07 μg ml^−1^ (isolate JZ-8) to 30.7 μg ml^−1^ (isolate Zang-8). Sixty-one isolates solubilized phosphate with halo diameter (HD) to the colony diameter (CD) values from 0.24 (strain JZ-5) to 2.67 (strain JZ-7). The HD/CD values of strains LZ-2, Zang-8, Zang-10, LZ6-9, Ali-3, and LZ-9 were all above 2.00. Fifty isolates produced cellulase with HD/CD values from 0.09 (isolate LZ4-10) to 3.12 (isolate LZ-9). The isolates JZ-7 (*Bacillus siamensis*), LZ-9 (*Bacillus tequilensis*), and Zang-8 (*Bacillus wiedmannii*) showed high siderophore production and N_2_ fixation ability (Supplementary Table S4) and were selected for the plant growth promotion ability test.

The plant growth promotion ability of *Bacillus* spp. isolates Zang8, JZ-7, and LZ6-9 was estimated in a pot experiment. Compared to the not-inoculated plants, inoculation with *B. tequilensis* LZ-9 resulted in greater length and number of roots, and in bigger aboveground and root fresh and dry weights; inoculation with *B. siamensis* JZ-7 resulted in smaller aboveground fresh weight and bigger root dry weight; and inoculation with *B. wiedmannii* Zang-8 resulted in greater plant height and leaf length, and in smaller number of roots and aboveground fresh weight (*p* < 0.05; Table 2).

**Table 2:** The effects of highland barley (HB) seed endophyte isolates on the growth of HB seedlings.

Inoculum	Plant height (cm)	Leaf length (cm)	Leaf width (cm)	Root length (cm)	Aboveground fresh weight (g)	Fresh root weight (g)	Aboveground dry weight (g)	Dry root weight (g)	Root number
CK	42.3 ± 0.46b	26.2 ± 2.54c	1.22 ± 0.038a	12.6 ± 1.35c	2.55 ± 0.393b	0.28 ± 0.037b	0.237 ± 0.035b	0.163 ± 0.019b	23.3 ± 0.98b
JZ-7	42.4 ± 0.22b	29.1 ± 0.97b	1.23 ± 0.064a	14.5 ± 0.49b	1.79 ± 0.009c	0.27 ± 0.009b	0.217 ± 0.003b	0.200 ± 0.008a	25.0 ± 1.41b
Zang-8	46.1 ± 0.85a	34.5 ± 0.71a	1.28 ± 0.027a	12.8 ± 1.82c	1.52 ± 0.028c	0.25 ± 0.019b	0.213 ± 0.014b	0.160 ± 0.009b	16.3 ± 0.72c
LZ-9	43.4 ± 1.11b	30.9 ± 1.16b	1.23 ± 0.086a	16.6 ± 1.42a	3.68 ± 0.443a	0.39 ± 0.036a	0.497 ± 0.009a	0.213 ± 0.027a	28.3 ± 1.44a

Data are mean value ± SE (*n* = 15). Different letters in a column denote statistically significant differences (*p* < 0.05). CK, uninoculated control.

### Endophytic bacterial communities

**Figure 4:**
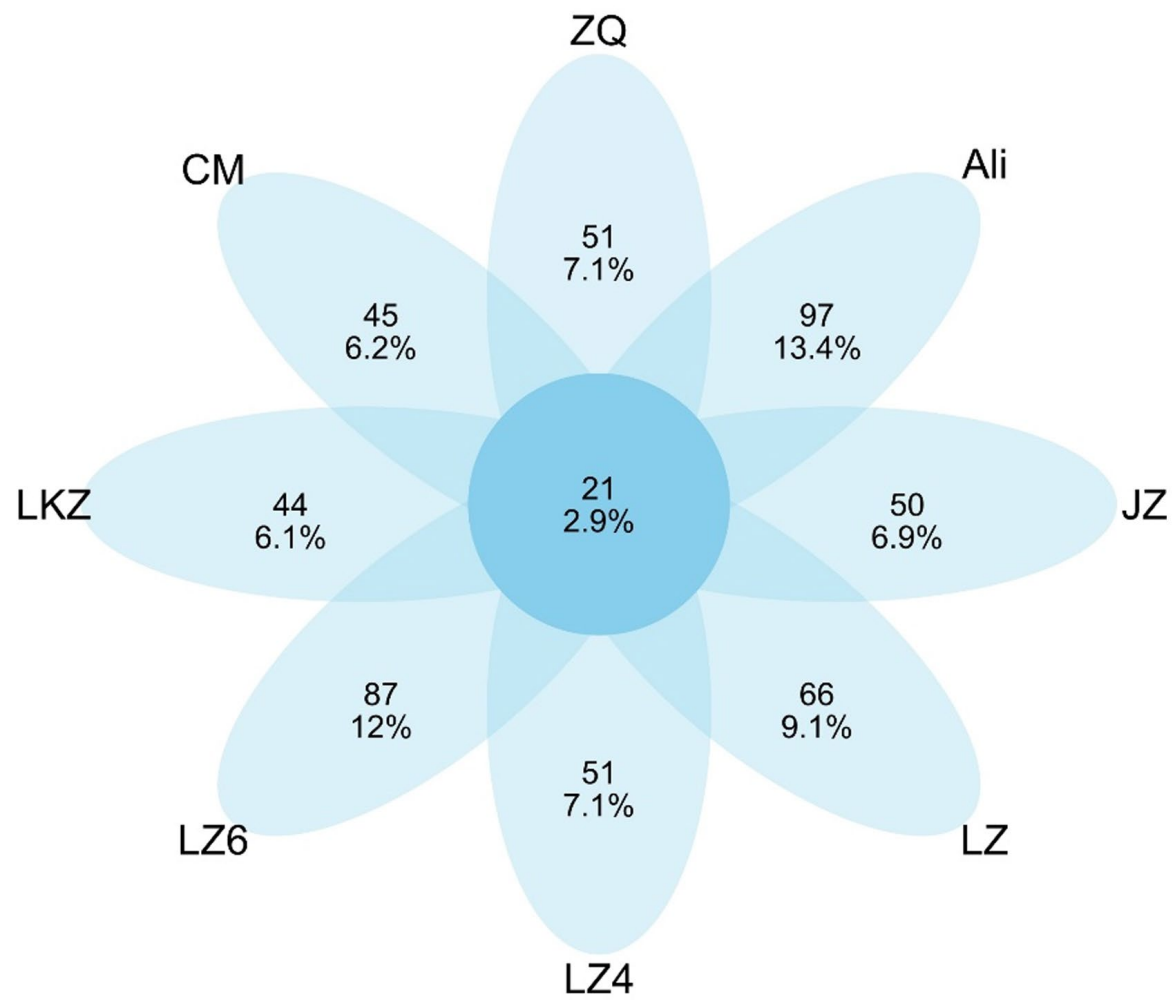
Shared and unique 16S rRNA gene amplicon sequence variants (ASVs) in the seeds of highland barley varieties. CM, Cuomei; JZ, Jiangzi; LZ, Lazi; LZ4, Longzi4ling; LZ6, Longzi6ling; LKZ, Langkazi; and ZQ, Zangqing 2000.

**Figure 5:**
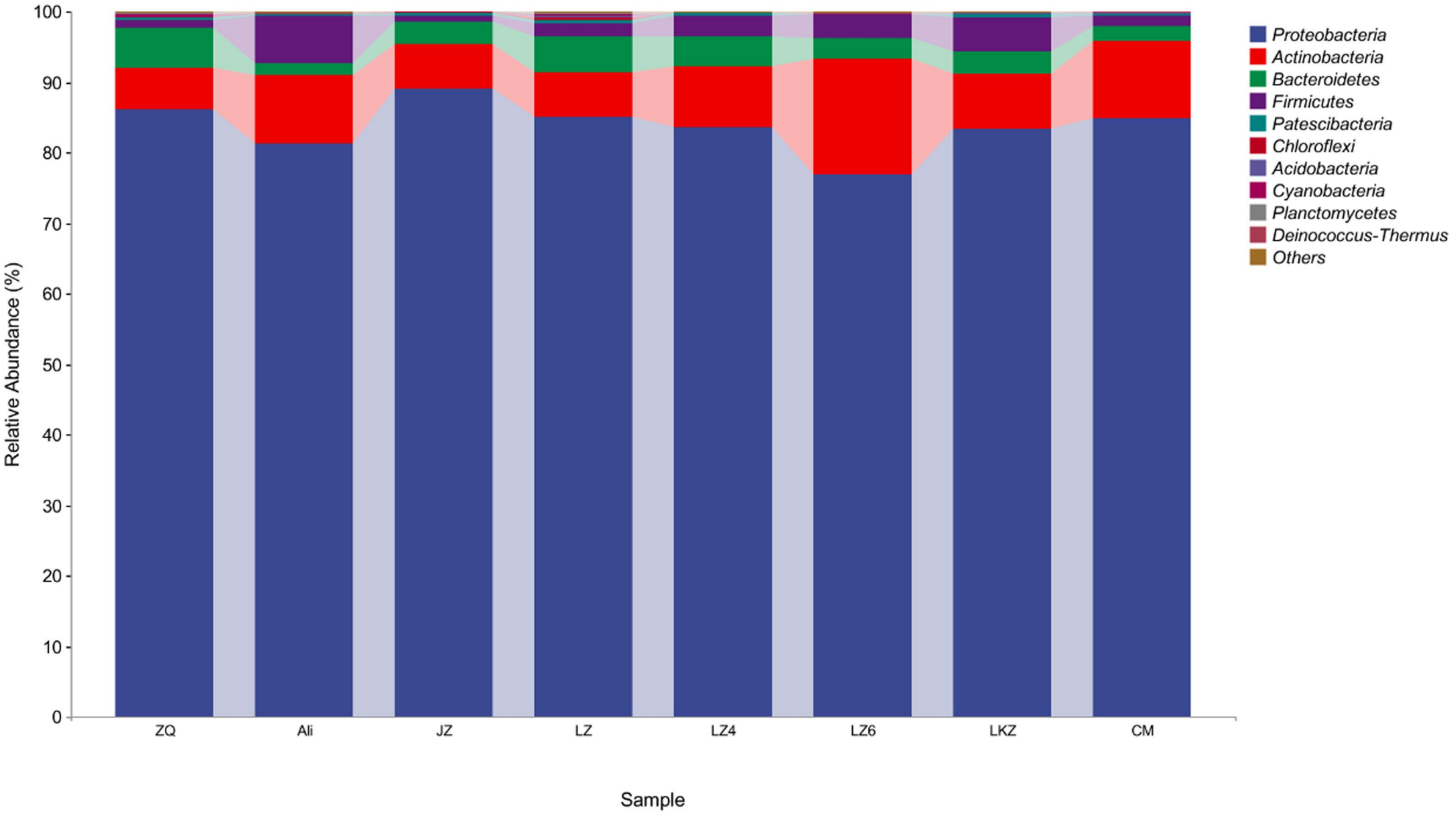
The phylum level relative abundances of the endophytes from the seeds of highland barley varieties. CM, Cuomei; JZ, Jiangzi; LZ, Lazi; LZ4, Longzi4ling; LZ6, Longzi6ling; LKZ, Langkazi; and ZQ, Zangqing 2000.

The endophytic bacterial communities in the seeds of the HB varieties were characterized using amplicon sequencing targeting the 16S rRNA gene. After removal of plant derived sequences and resampling, the remaining 8,832 sequences were clustered into 722 ASVs. Only 21 of the ASVs were detected in all the HB varieties, and from 44 to 97 ASVs were unique to the varieties (Figure 4). The mean relative abundance of Proteobacteria was 83.8% (Figure 5). The relative abundances of phyla Actinobacteria, Bacteroidetes, and Firmicutes ranged from 5.8 to 16.4%, 1.7 to 5.8%, and 0.9 to 6.7%, respectively (Figure 5). At the genus level, the relative abundance of proteobacterial ASVs assigned into *Pseudomonas*, *Halomonas*, *Pantoea*, and *Pelagibacterium* was from 43.3 to 59.0% of the total abundance (Supplementary Figure S1).

**Figure 6:**
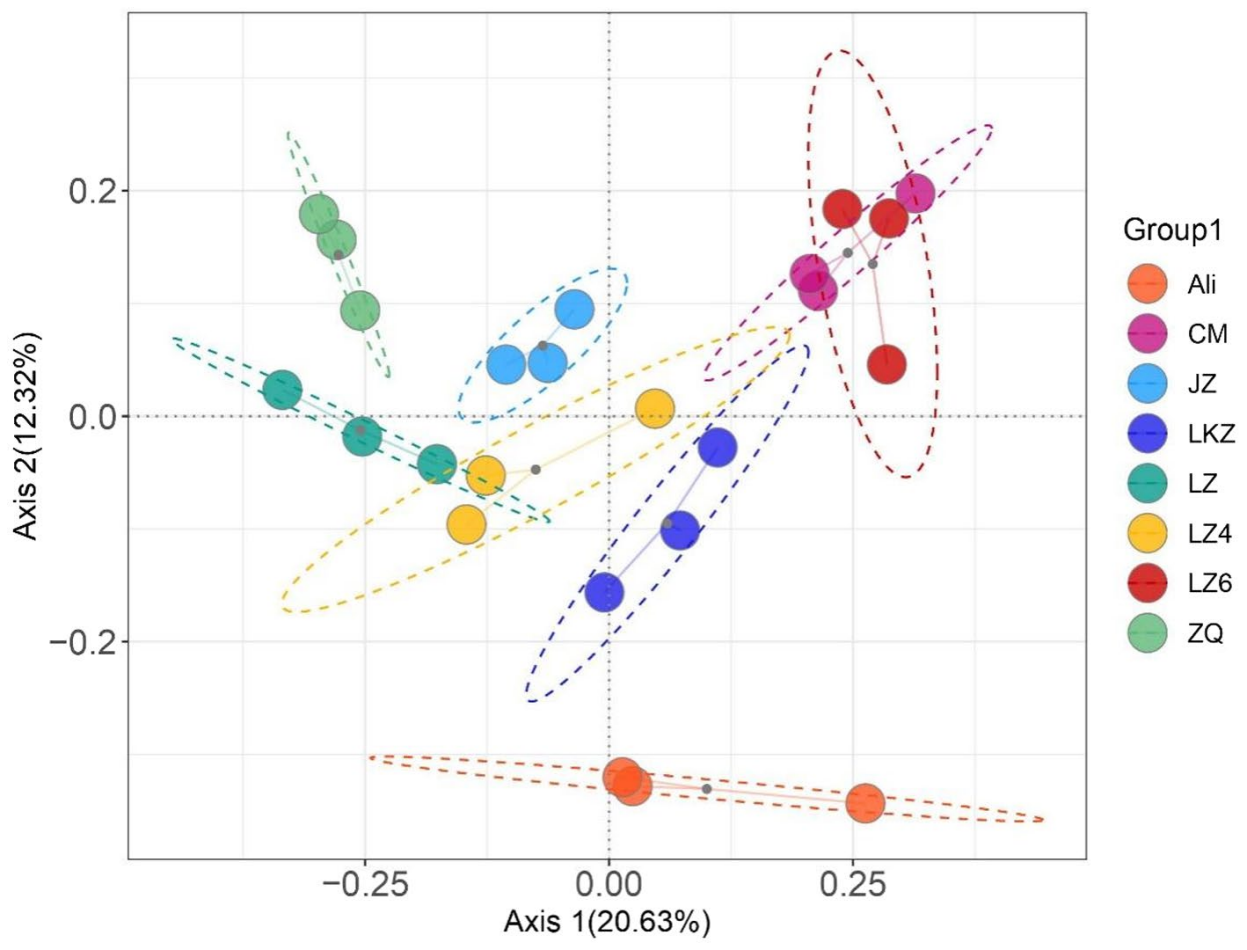
Principal coordinates analysis of the endophytic bacterial communities in the seeds of highland barley varieties. CM, Cuomei; JZ, Jiangzi; LZ, Lazi; LZ4, Longzi4ling; LZ6, Longzi6ling; LKZ, Langkazi; and ZQ, Zangqing 2000.

Both the richness and diversity varied among the HB seed bacterial communities (*p* < 0.05); the number of observed species ranged from 71.0 ± 4.97 to 121 ± 2.81, and the Shannon index from 5.05 ± 0.333 to 6.08 ± 0.024 (Supplementary Table S5). Overall, the bacterial community compositions in the different HB varieties were different from each other (PERMANOVA pseudo-*F* = 3.45, *p* = 0.001; Figure 6; Supplementary Table S6). No statistically significant differences were found in the pairwise PERMANOVA, suggesting that the between variety differences in community composition were relatively small.

**Figure 7:**
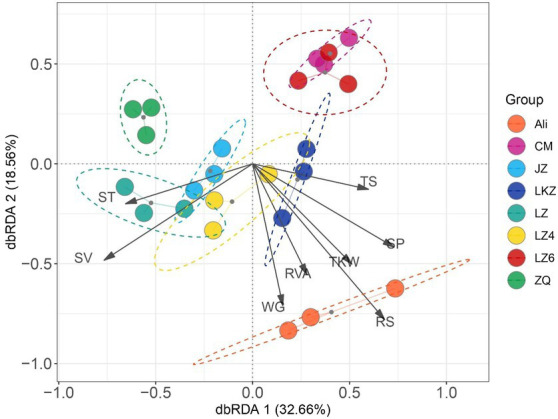
The relationships between the highland barley seed properties and the endophytic bacterial communities in the seeds of highland barley varieties. The abbreviations of highland barley varieties are as in Figure 1. AM, amylose content; TS, total sugars; RS, reducing sugars; WG, wet gluten; SV, sedimentation value; SP, soluble protein; RVA, viscosity; and TKW, 1000-kernel weight.

The relationships between the properties of the HB varieties and the bacterial communities were assessed using distance-based redundancy analysis (dbRDA) and Mantel tests. The endophytic communities in the eight HB varieties were not clearly separated along the dbRDA axis 1 that explained 32.66% of the total variability (Figure 7). The communities in the variety Ali were separated from the other communities along axis 2. Both the Mantel and Partial-Mantel analysis showed that the differences in bacterial communities were associated with differences in reducing sugar content (RS), viscosity (RVA), and total sugar content (TS; Supplementary Figures S2, S3).

## Discussion

Plants are associated with multiple micro-organisms that play important roles in their growth and development. We studied endophytic bacteria in the seeds of highland barley (HB), an economically important crop and a major part of the local diet in the Tibetan Plateau, China, to characterize the seed bacterial communities and assess their plant growth promoting (PGP) potential.

Most of the 86 cultured isolates were identified as *Bacillus* spp., and the rest as *Alcaligenes aquatilis*, *Proteus alimentorum*, and *Enterobacter* spp. *Bacillus* endophytes species have been widely identified and characterized as producers of growth-promoting substances, bioactive compounds, and metabolites with antimicrobial effects (Lopes et al., 2018). *Bacillus* species also hold immense agricultural development potential because of their ability to form endospores with long shelf lives, resistance against heat exposure and desiccation (Chowdhury et al., 2013). The *Bacillus* species like *B. amyloliquefaciens*, *B. licheniformis,* and *B. subtilis* have been proven to be effective in plant growth promotion and *Bacillus* products are available as biofertilizers (Pérez-García et al., 2011; Hashem et al., 2019). Interestingly, similar to rice seeds (Walitang et al., 2018), the relative abundance of *Bacillus* 16S rRNA gene amplicons was low in the highland barley seeds, possibly due to a cultivation bias that leads to over-representation of *Firmicutes* and *Proteobacteria* in cultivation-based analyses (Overmann et al., 2017). The culture dependent and independent approaches are complementary since culture-independent methods give a wider view the endophytic communities (Hardoim et al., 2008). The knowledge of the wide spectrum of HB seed endophytic bacteria will facilitate the search of bacteria capable of promoting the growth of HB. A more comprehensive view of HB seed endophytic bacteria could be obtained *via* metagenomic and functional metagenomic analyses, and the results from culture independent analyses like in this study provide a basis for the future studies.

The ɣ-*Proteobacteria* in the HB seed endophyte communities were majorly affiliated with the *Enterobacteriaceae* family. *Enterobacteriaceae* were the dominant seed endophytes in seven barley accessions (Bziuk et al., 2021) and prevailed in plant-associated microbiomes, e.g., lettuce (*Lactuca sativa* L.), arugula (*Eruca sativa* Mill.), orchard grass (*Dactylis glomerata* L.), passion flower (*Passiflora incarnate* L.), and tomato (*Solanum lycopersicum* cv. Hawaii 7996; Estendorfer et al., 2017; Cernava et al., 2019; Roy et al., 2019). *Enterobacteriaceae* include diverse and versatile members with plant growth promoting characteristics both *in vitro* and *in vivo* (Jeong et al., 2021). In our study, the most dominant *Enterobacteriaceae* genus was *Pantoea*. Although species like *Pantoea agglomerans* and *P. ananatis* have been implicated as plant pathogens causing plant rot, blight, and dieback (De Maayer et al., 2010), *P. ananatis* and *P. agglomerans* include plant growth promoting strains (Walterson and Stavrinides, 2015).

The relative abundance of genus *Halomonas* in ɣ-*Proteobacteria* was high in all the eight highland barley varieties. *Halomonas*, a phenotypically heterogeneous gram-negative genus, can grow in 5–25% NaCl and survive in nitrate-containing environment, showing a high level of denitrification activity (Mata et al., 2002; Zhu et al., 2012). *Halomonas* endophytes have been isolated from roots of halophytes, such as *Salicornia rubra*, *Sarcocornia utahensis*, and *Allenrolfea occidentalis* (Kearl et al., 2019). *Halomonas* and *Kushneria* are closely related, and in the past were grouped in the same genus (Sanchez-Porro et al., 2009). *Kushneria* strains have been mostly isolated from saline environments (Yun et al., 2018) and from the endosphere and phyllosphere of halophyte plants (Bangash et al., 2015; Navarro-Torre et al., 2018). Members of the genus *Halomonas* and *Kushneria* have shown plant growth-promoting activities, including siderophore production, indolacetic acid (IAA) production, nitrogen fixation, and phosphate solubilization (Navarro-Torre et al., 2016; Kearl et al., 2019). To our knowledge, this was the first time *Halomonas* were detected in high relative abundance in highland barley seeds, implying that *Halomonas* may enhance the tolerance of highland barley against the ongoing soil salinization in Qinghai-Tibet region caused by the change of global climate in recent years (Li et al., 2012; Chang et al., 2021).

Although the relationships between host plant genotype and environment and the diversity of seed endophytic bacteria (SEB) communities have received considerable attention, the relations between seed endophytic bacterial community and the properties of seeds have been seldom studied. Moreover, results on the role of the host plant genotype in shaping the composition of SEB community have been inconsistent. Our results showed that the differences in SEB microbiomes were associated with the total and reducing sugar contents and viscosity, a parameter determined by the starch content and composition, all of which are connected to osmotic pressure inside the seed. In rice seeds, the accumulation of carbohydrates during the seed maturation increased the osmotic pressure and selected endospore forming and high osmotic pressure tolerant strains (Mano et al., 2006). Possibly an analogous process governed the HB seed endophytes, i.e., the community level differences were connected to differences in tolerance against osmotic pressure. Johnston-Monje et al. (2014) found that the SEB community composition in maize seeds was mainly shaped by the maize genotype. The seed microbiomes in seven barley accessions were host plant genotype-dependent, yet all the accessions shared a core microbiome with high relative abundance (Bziuk et al., 2021). In our study, the differences between SEB communities were minor even though the eight highland barley varieties varied significantly based on the ISSRs molecular marker analysis. Plausibly, the high abundance core microbiome in HB seeds masked the between variety differences.

## Conclusion

We characterized endophytic bacteria in the seeds of eight highland barley varieties using cultivation and 16S rRNA targeting amplicon sequencing. Most of the isolates were assigned into genus *Bacillus* and approximately half of the isolates showed plant growth-promoting characteristics, suggesting that the seed endophytes of highland barley are likely to promote the growth since germination. Based on the 16S rRNA gene sequencing, the seed microbiome was majorly affiliated with the phylum Proteobacteria and the family *Enterobacteriaceae*. The community level differences between highland barley varieties were possibly connected to differences in tolerance against osmotic pressure.

## Data availability statement

The datasets presented in this study can be found in online repositories. The names of the repository/repositories and accession number(s) can be found in the article/Supplementary material.

## Author contributions

YG, TN, and PP conceived and designed research. YC, AZ, and JL analyzed the data and wrote the manuscript. XG and YW collected the seed samples. LZ, QX, and QC collected literatures. KZ and XY analyzed the seed samples and data. All authors contributed to the article and approved the submitted version.

## Funding

This study was supported by the Projects of Local Funds under the Guidance of the Central Government of China (grant no. Y20X20195400004790), the National Natural Science Foundation of China (grant no. 41201256), and Sichuan Agricultural University (grant no. 1921993363).

## Conflict of interest

The authors declare that the research was conducted in the absence of any commercial or financial relationships that could be construed as a potential conflict of interest.

## Publisher’s note

All claims expressed in this article are solely those of the authors and do not necessarily represent those of their affiliated organizations, or those of the publisher, the editors and the reviewers. Any product that may be evaluated in this article, or claim that may be made by its manufacturer, is not guaranteed or endorsed by the publisher.

## Supplementary material

The Supplementary material for this article can be found online at: https://www.frontiersin.org/articles/10.3389/fmicb.2022.981158/full#supplementary-material

## Footnotes

^1^ https://www.ncbi.nlm.nih.gov/sra/

^2^ https://docs.qiime2.org/2019.4/tutorials/

## References

[ref1] AACC (2000). Approved methods of the AACC International, methods 44–17, 76–13, 08–16, 32–40, 61–02, and 35–05 (10th Edn.), St. Paul, MN: The Association AACC.

[ref2] Bangash A. Ahmed I. Abbas S. Kudo T. Shahzad A. Fujiwara T.. (2015). Kushneria pakistanensis sp. nov., a novel moderately halophilic bacterium isolated from rhizosphere of a plant (*Saccharum spontaneum*) growing in salt mines of the Karak area in Pakistan. Antonie Van Leeuwenhoek 107, 991–1000. doi: 10.1007/s10482-015-0391-9, PMID: 25631404

[ref3] Bokulich N. A. Kaehler B. D. Rideout J. R. Dillon M. Bolyen E. Knight R.. (2018). Optimizing taxonomic classification of marker-gene amplicon sequences with QIIME 2’s q2-feature-classifier plugin. Microbiome 6:90. doi: 10.1186/s40168-018-0470-z, PMID: 29773078 PMC5956843

[ref4] Bziuk N. Maccario L. Straube B. Wehner G. Sørensen S. J. Schikora A.. (2021). The treasure inside barley seeds: microbial diversity and plant beneficial bacteria. Environ. Microbiol. 16:20. doi: 10.1186/S40793-021-00389-8, PMID: 34711269 PMC8554914

[ref5] Caporaso J. G. Lauber C. L. Walters W. A. Berg-Lyons D. Huntley J. Fierer N.. (2012). Ultra-high-throughput microbial community analysis on the Illumina HiSeq and MiSeq platforms. ISME J. 6, 1621–1624. doi: 10.1038/ismej.2012.8, PMID: 22402401 PMC3400413

[ref6] Cernava T. Erlacher A. Soh J. Sensen C. W. Grube M. Berg G. (2019). Enterobacteriaceae dominate the core microbiome and contribute to the resistome of arugula (*Eruca sativa* mill.). Microbiome 7:13. doi: 10.1186/s40168-019-0624-7, PMID: 30696492 PMC6352427

[ref7] Chang Y. X. Zhang J. T. Bao G. Z. Yan B. R. Qu Y. Zhang M. Y.. (2021). Physiological responses of highland barley seedlings to NaCl, drought, and freeze-thaw stress. J. Plant Growth Regul. 40, 154–161. doi: 10.1007/s00344-020-10085-5

[ref8] Chowdhury S. P. Dietel K. Rändler M. Schmid M. Junge H. Borriss R.. (2013). Effects of *Bacillus amyloliquefaciens* fzb42 on lettuce growth and health under pathogen pressure and its impact on the rhizosphere bacterial community. PLoS One 8:e68818. doi: 10.1371/journal.pone.0068818, PMID: 23935892 PMC3720850

[ref9] De Maayer P. Chan W. Y. Venter S. N. Toth I. K. Birch P. R. Joubert F.. (2010). Genome sequence of *Pantoea ananatis* LMG20103, the causative agent of Eucalyptus blight and dieback. J. Bacteriol. 192, 2936–2937. doi: 10.1128/JB.00060-10, PMID: 20348253 PMC2876505

[ref10] DeLong E. F. (1992). Archaea in coastal marine environments. PNAS 89, 5685–5689. doi: 10.1073/pnas.89.12.5685, PMID: 1608980 PMC49357

[ref11] Deng J. Ma X. Zhao T. Yi J. Liu X. (2018). Effect of highland barley glucan on starch digestibility in vitro. Food Sci. 39, 106–111. doi: 10.7506/spkx1002-6630-201810017

[ref12] DeSantis T. Z. Hugenholtz P. Larsen N. Rojas M. Andersen G. L. (2006). Greengenes, a chimera-checked 16S rRNA gene database and workbench compatible with ARB. Appl. Environ. Microbiol. 72, 5069–5072. doi: 10.1128/AEM.03006-05, PMID: 16820507 PMC1489311

[ref13] Döbereiner J. Baldani V.L.D. Baldani J.L. (1995). Como Isolar e Identificar Bactérias Diazotróficas de Plantas Não-Leguminosas. Brasília: Embrapa-SPI

[ref14] Durand A. Leglize P. Benizri E. (2021). Are endophytes essential partners for plants and what are the prospects for metal phytoremediation? Plant Soil 460, 1–30. doi: 10.1007/s11104-020-04820-w

[ref15] Edwards J. Johnson C. Santos-Medellín C. Lurie E. Sundaresan V. (2015). Structure, variation, and assembly of the root-associated microbiomes of rice. PNAS 112, E911–E920. doi: 10.1073/pnas.1414592112, PMID: 25605935 PMC4345613

[ref16] Eevers N. Gielen M. Sánchez-López A. Jaspers S. White J. C. Vangronsveld J.. (2015). Optimization of isolation and cultivation of bacterial endophytes through addition of plant extract to nutrient media. Microb. Biotechnol. 8, 707–715. doi: 10.1111/1751-7915.12291, PMID: 25997013 PMC4476825

[ref17] Estendorfer J. Stempfhuber B. Haury P. Vestergaard G. Rillig M. C. Joshi J.. (2017). The influence of land use intensity on the plant-associated microbiome of *Dactylis glomerata* L. Front. Plant Sci. 8:930. doi: 10.3389/fpls.2017.00930, PMID: 28680426 PMC5478725

[ref18] Gao W. Gong L. Zhang Y. (2015). The development potential of Tibetan hull-less barley as China plateau characteristic grain resource. J. Cereals Oils 28, 1–4.

[ref19] Gitaitis R. Walcott R. (2007). The epidemiology and management of seedborne bacterial diseases. Annu. Rev. Phytopathol. 45, 371–397. doi: 10.1146/annurev.phyto.45.062806.094321, PMID: 17474875

[ref20] Glickmann E. Dessaux Y. (1995). A critical examination of the specificity of the Salkowski reagent for indolic compounds produced by phytopathogenic bacteria. Appl. Environ. Microbiol. 61, 793–796. doi: 10.1128/aem.61.2.793-796.19, PMID: 16534942 PMC1388360

[ref22] Grumet R. Gifford F. (1998). Plant biotechnology in the United States: issues and challenges en route to commercial production. HortScience 33, 187–192. doi: 10.1023/A:1008693614060

[ref24] Guo J. Bowatte S. Hou F. (2021). Diversity of endophytic bacteria and fungi in seeds of Elymus nutans growing in four locations of Qinghai-Tibet plateau, China. Plant Soil 459, 49–63. doi: 10.1007/s11104-020-04608-y

[ref25] Guo T. Horvath C. Chen L. Chen J. Zheng B. (2020). Understanding the nutrient composition and nutritional functions of highland barley (Qingke): a review. Trends Food Sci. Technol. 103, 109–117. doi: 10.1016/j.tifs.2020.07.011

[ref26] Hardoim P. R. van Overbeek L. S. van Elsas J. D. V. (2008). Properties of bacterial endophytes and their proposed role in plant growth. Trends Microbiol. 16, 463–471. doi: 10.1016/j.tim.2008.07.008, PMID: 18789693

[ref27] Hardy R. W. F. Holsten R. D. Jackson E. K. Burns R. C. (1968). The acetylene-ethylene assay for N_2_ fixation-laboratory and field evaluation. Plant Physiol. 43, 1185–1207. doi: 10.1104/pp.43.8.1185, PMID: 16656902 PMC1086994

[ref28] Hashem A. Tabassum B. Abd Allah E. F. (2019). *Bacillus subtilis*: a plant-growth promoting rhizobacterium that also impacts biotic stress. Saudi J. Biol. Sci. 26, 1291–1297. doi: 10.1016/j.sjbs.2019.05.004, PMID: 31516360 PMC6734152

[ref29] Healey A. Furtado A. Cooper T. Henry R. J. (2014). Protocol: a simple method for extracting next-generation sequencing quality genomic DNA from recalcitrant plant species. Plant Methods 10:21. doi: 10.1186/1746-4811-10-21, PMID: 25053969 PMC4105509

[ref30] Huang S. Pang F. (2017). Biocontrol agents for controlling wheat rust. Methods Mol. Biol. 1659, 277–288. doi: 10.1007/978-1-4939-7249-4_24, PMID: 28856659

[ref31] Jeong S. Kim T. M. Choi B. Kim Y. Kim E. (2021). Invasive *Lactuca serriola* seeds contain endophytic bacteria that contribute to drought tolerance. Sci. Rep. 11:13307. doi: 10.1038/S41598-021-92706-X, PMID: 34172799 PMC8233371

[ref32] Johnston-Monje D. Mousa W. K. Lazarovits G. Raizada M. N. (2014). Impact of swapping soils on the endophytic bacterial communities of pre-domesticated, ancient and modern maize. BMC Plant Biol. 14:233. doi: 10.1186/s12870-014-0233-3, PMID: 25227492 PMC4189167

[ref33] Kaga H. Mano H. Tanaka F. Watanabe A. Kaneko S. Morisaki H. (2009). Rice seeds as sources of endophytic bacteria. Microbes Environ. 24, 154–162. doi: 10.1264/jsme2.ME09113, PMID: 21566368

[ref34] Katoh K. Misawa K. Kuma K. Miyata T. (2002). MAFFT: a novel method for rapid multiple sequence alignment based on fast Fourier transform. Nucleic Acids Res. 30, 3059–3066. doi: 10.1093/nar/gkf436, PMID: 12136088 PMC135756

[ref35] Kearl J. Mcnary C. Lowman J. S. Mei C. Nielsen B. L. (2019). Salt-tolerant halophyte rhizosphere bacteria stimulate growth of alfalfa in salty soil. Front. Microbiol. 10:1849. doi: 10.3389/fmicb.2019.01849, PMID: 31474952 PMC6702273

[ref36] Langfelder P. Horvath S. (2008). WGCNA: an R package for weighted correlation network analysis. BMC Bioinformatics 9:559. doi: 10.1186/1471-2105-9-559, PMID: 19114008 PMC2631488

[ref37] Legendre P. Gallagher E. D. (2001). Ecologically meaningful transformations for ordination of species data. Oecologia 129, 271–280. doi: 10.1007/s004420100716, PMID: 28547606

[ref38] Li J. Pu L. Zhu M. Zhang R. (2012). The present situation and hot issues in the salt-affected soil research. J. Geogr. Sci. 67, 1233–1245. doi: 10.11821/xb201209008

[ref39] Liu Z. W. Biyashev R. M. Maroof M. A. S. (1996). Development of simple sequence repeat DNA markers and their integration into a barley linkage map. Theor. Appl. Genet. 93, 869–876. doi: 10.1007/BF00224088, PMID: 24162420

[ref40] Lopes R. Tsui S. Gonçalves P. J. R. O. de Queiroz M. V. (2018). A look into a multifunctional toolbox: endophytic Bacillus species provide broad and underexploited benefits for plants. World J. Microbiol. Biotechnol. 34:94. doi: 10.1007/s11274-018-2479-7, PMID: 29900507

[ref41] Mano H. Tanaka F. Watanabe A. Kaga H. Okunishi S. Morisaki H. (2006). Culturable surface and endophytic bacterial flora of the maturing seeds of rice plants (*Oryza sativa*) cultivated in a paddy field. Microbes Environ. 21, 86–100. doi: 10.1264/jsme2.21.86

[ref42] Martin M. (2011). Cutadapt removes adapter sequences from high-throughput sequencing reads. EMBnet J. 17, 10–12. doi: 10.14806/ej.17.1.200

[ref43] Mata J. A. Martínez-Cánovas J. Quesada E. Béjar V. (2002). A detailed phenotypic characterisation of the type strains of halomonas species. Syst. Appl. Microbiol. 25, 360–375. doi: 10.1078/0723-2020-00122, PMID: 12421074

[ref44] Mitter B. Pfaffenbichler N. Flavell R. Compant S. Antonielli L. Petric A.. (2017). A new approach to modify plant microbiomes and traits by introducing beneficial bacteria at flowering into progeny seeds. Front. Microbiol. 8:11. doi: 10.3389/fmicb.2017.00011, PMID: 28167932 PMC5253360

[ref45] Mora-Ruiz M. R. Francisca F. V. Alejandro O. Joan R. Ramon R. M. (2016). Endophytic microbial diversity of the halophyte Arthrocnemum macrostachyum across plant compartments. FEMS Microbiol. Ecol. 92:fiw145. doi: 10.1093/FEMSEC/FIW145 27353659

[ref46] Navarro-Torre S. Carro L. Rodríguez-Llorente I. D. Pajuelo E. Montero-Calasanz M. D. C. (2018). Kushneria phyllosphaerae sp. nov. and kushneria endophytica sp. nov. plant growth promoting endophytes isolated from the halophyte plant Arthrocnemum macrostachyum. Int. J. Syst. Evol. Microbiol. 68, 2800–2806. doi: 10.1099/ijsem.0.002897, PMID: 30010522

[ref47] Navarro-Torre S. Mateos-Naranjo E. Caviedes M. A. Pajuelo E. Rodríguez-Llorente I. D. (2016). Isolation of plant-growth-promoting and metal-resistant cultivable bacteria from Arthrocnemum macrostachyum in the odiel marshes with potential use in phytoremediation. Mar. Pollut. Bull. 110, 133–142. doi: 10.1016/j.marpolbul.2016.06.070, PMID: 27349383

[ref48] Nelson E. B. (2004). Microbial dynamics and interactions in the spermosphere. Annu. Rev. Phytopathol. 42, 271–309. doi: 10.1146/annurev.phyto.42.121603.131041, PMID: 15283668

[ref49] Obadi M. Sun J. Xu B. (2021). Highland barley: chemical composition, bioactive compounds, health effects, and applications. Food Res. Int. 140:110065. doi: 10.1016/j.foodres.2020.110065, PMID: 33648288

[ref50] Oksanen J Kindt R Pierre L O'Hara B Simpson GL Solymos P. (2017). Vegan: Community Ecology Package. R Package Version 2.4–4. Available at: http://CRAN.R-project.org/package=vegan

[ref52] Overmann J. Abt B. Sikorski J. (2017). Present and future of culturing bacteria. Annu. Rev. Microbiol. 71, 711–730. doi: 10.1146/annurev-micro-090816-093449, PMID: 28731846

[ref53] Pérez-García A. Romero D. Vicente A. D. (2011). Plant protection and growth stimulation by microorganisms: biotechnological applications of Bacilli in agriculture. Curr. Opin. Biotechnol. 22, 187–193. doi: 10.1016/j.copbio.2010.12.003, PMID: 21211960

[ref54] Pikovskaya R. I. (1948). Mobilization of phosphorus in soil in connection with the vital activity of some microbial species. Mikrobiologiya 17, 362–370.

[ref55] Rahman M. M. Flory E. Koyro H. W. Abideen Z. Schikora A. Suarez C.. (2018). Consistent associations with beneficial bacteria in the seed endosphere of barley (*Hordeum vulgare* L.). Syst. Appl. Microbiol. 41, 386–398. doi: 10.1016/j.syapm.2018.02.003, PMID: 29567394

[ref56] Rios-Galicia B. Villagómez-Garfias C. De la Vega-Camarillo E. Guerra-Camacho J. E. Medina-Jaritz N. Arteaga-Garibay R. I.. (2021). The Mexican giant maize of Jala landrace harbour plant-growth-promoting rhizospheric and endophytic bacteria. 3 Biotech 11:447. doi: 10.1007/S13205-021-02983-6, PMID: 34631348 PMC8463652

[ref57] Roy N. Choi K. Khan R. Lee S. W. (2019). Culturing simpler and bacterial wilt suppressive microbial communities from tomato rhizosphere. Plant Pathol. J. 35, 362–371. doi: 10.5423/PPJ.FT.07.2019.0180, PMID: 31481859 PMC6706014

[ref58] Sánchez-López A. S. Pintelon I. Stevens V. Imperato V. Timmermans J. P. González-Chávez C.. (2018). Seed endophyte microbiome of *Crotalaria pumila* unpeeled: identification of plant-beneficial methylobacteria. Int. Mol. Sci. 19:291. doi: 10.3390/ijms19010291, PMID: 29351192 PMC5796236

[ref59] Sanchez-Porro C. De L. Soto-Ramirez N. Marquez M. C. Montalvo-Rodriguez R. Ventosa A. (2009). Description of *Kushneria aurantia* gen. Nov. sp. nov. a novel member of the family Halomonadaceae, and a proposal for reclassification of *Halomonas marisflavi* as *Kushneria marisflavi* comb. nov. of *Halomonas indalinina* as *Kushneria indalinina* comb. nov. a. Int. J. Syst. Evol. Microbiol. 59, 397–405. doi: 10.1099/ijs.0.001461-0 19196785

[ref60] Schuler C. J. Hirsch M. Harmeling S. Schoelkopf B. (2016). Learning to deblur. IEEE Trans. Pattern Anal. Mach. Intell. 38, 1439–1451. doi: 10.1109/TPAMI.2015.2481418, PMID: 26415157

[ref61] Schwyn B. Nielands J. B. (1987). Universal chemical assay for the detection and determination of siderophores. Anal. Biochem. 160, 47–56. doi: 10.1016/0003-2697(87)90612-9, PMID: 2952030

[ref62] Slowikowski K. Schep A. Hughes S. Lukauskas S. Irisson J.O. Kamvar Z.N.. (2018). ggrepel: Automatically Position Non-Overlapping Text Labels with ‘ggplot2’. Available at: https://CRAN.R-project.org/package=ggrepel

[ref63] Tamura K. Stecher G. Peterson D. Filipski A. Kumar S. (2013). MEGA6: molecular evolutionary genetics analysis version 6.0. Mol. Biol. Evol. 30, 2725–2729. doi: 10.1093/molbev/mst197, PMID: 24132122 PMC3840312

[ref64] Teather R. M. Wood P. J. (1982). Use of Congo red-polysaccharide interactions in enumeration and characterization of cellulolytic bacteria from the bovine rumen. Appl. Environ. Microbiol. 43, 777–780. doi: 10.1111/j.1749-6632.1994.tb44394.x, PMID: 7081984 PMC241917

[ref65] Thomas H. James A. R. (1999). Partitioning of sugars in *Lolium perenne* (perennial ryegrass) during drought and on rewatering. New Phytol. 142, 295–305. doi: 10.1046/j.1469-8137.1999.00388.x

[ref66] Truyens W. Cuypers V. (2015). Bacterial seed endophytes: genera, vertical transmission and interaction with plants. Environ. Microbiol. Rep. 7, 40–50. doi: 10.1111/1758-2229.12181

[ref67] Vandenkoornhuyse P. Quaiser A. Duhamel M. Le Van A. Dufresne A. (2015). The importance of the microbiome of the plant halobiont. New Phytol. 206, 1196–1206. doi: 10.1111/nph.13312 25655016

[ref68] Vaneechoutte M. Rossau R. De Vos P. Gillis M. Janssens D. Paepe N.. (1992). Rapid identification of bacteria of the Comamonadaceae with amplified ribosomal DNA-restriction analysis (ARDRA). FEMS Microbiol. Lett. 72, 227–233. doi: 10.1111/j.1574-6968.1992.tb05102.x, PMID: 1354195

[ref69] Verma S. K. Kingsley K. Irizarry I. Bergen M. Kharwar R. N. White J. F. Jr. (2017). Seed-vectored endophytic bacteria modulate development of rice seedlings. J. Appl. Microbiol. 122, 1680–1691. doi: 10.1111/jam.13463, PMID: 28375579

[ref70] Vogl A. (1989). Mehl und die anderen mehl produkte der cerealien und leguminosen. Zeitschrift Nahrungsmittle untersuchung, Hgg Warlenkunde 21, 25–29.

[ref71] Walitang D. I. Kim C. G. Kim K. Kang Y. Y. Kim Y. K. Sa T. (2018). The influence of host genotype and salt stress on the seed endophytic community of salt-sensitive and salt-tolerant rice cultivars. BMC Plant Biol. 18:51. doi: 10.1186/s12870-018-1261-1, PMID: 29587643 PMC5870378

[ref72] Walterson A. M. Stavrinides J. (2015). Pantoea: insights into a highly versatile and diverse genus within the Enterobacteriaceae. FEMS Microbiol. Rev. 39, 968–984. doi: 10.1093/femsre/fuv027, PMID: 26109597

[ref74] Wang W. Zhai Y. Cao L. Tan H. Zhang R. (2016). Endophytic bacterial and fungal microbiota in sprouts, roots and stems of rice (*Oryza sativa* L.). Microbiol. Res. 188-189, 1–8. doi: 10.1016/j.micres.2016.04.009, PMID: 27296957

[ref77] Yun J. H. Sung H. Kim H. S. Tak E. J. Kang W. Lee J.-Y.. (2018). Complete genome sequence of the halophile bacterium *Kushneria konosiri* X49T, isolated from salt-fermented *Konosirus punctatus*. Stand. Genomic Sci. 13:19. doi: 10.1016/j.margen.2017.11.002, PMID: 30305867 PMC6167781

[ref78] Zhu L. Ding W. Feng L. J. Kong Y. Xu J. Xu X. Y. (2012). Isolation of aerobic denitrifiers and characterization for their potential application in the bioremediation of oligotrophic ecosystem. Bioresour. Technol. 108, 1–7. doi: 10.1016/j.biortech.2011.12.033, PMID: 22265979

